# Phosphorylation of Sli15 by Ipl1 Is Important for Proper CPC Localization and Chromosome Stability in *Saccharomyces cerevisiae*

**DOI:** 10.1371/journal.pone.0089399

**Published:** 2014-02-18

**Authors:** Vasso Makrantoni, Stephen J. Corbishley, Najma Rachidi, Nicholas A. Morrice, David A. Robinson, Michael J. R. Stark

**Affiliations:** 1 Centre for Gene Regulation and Expression, College of Life Sciences, University of Dundee, Scotland, United Kingdom; 2 MRC Protein Phosphorylation Unit, College of Life Sciences, University of Dundee, Scotland, United Kingdom; 3 Division of Biological Chemistry and Drug Discovery, College of Life Sciences, University of Dundee, Scotland, United Kingdom; University of Connecticut, Storrs, United States of America

## Abstract

The chromosomal passenger complex (CPC) is a key regulator of eukaryotic cell division, consisting of the protein kinase Aurora B/Ipl1 in association with its activator (INCENP/Sli15) and two additional proteins (Survivin/Bir1 and Borealin/Nbl1). Here we have identified multiple sites of CPC autophosphorylation on yeast Sli15 that are located within its central microtubule-binding domain and examined the functional significance of their phosphorylation by Ipl1 through mutation of these sites, either to non-phosphorylatable alanine (*sli15-20A*) or to acidic residues to mimic constitutive phosphorylation (*sli15-20D*). Both mutant *sli15* alleles confer chromosome instability, but this is mediated neither by changes in the capacity of Sli15 to activate Ipl1 kinase nor by decreased efficiency of chromosome biorientation, a key process in cell division that requires CPC function. Instead, we find that mimicking constitutive phosphorylation of Sli15 on the Ipl1 phosphorylation sites causes delocalization of the CPC in metaphase, whereas blocking phosphorylation of Sli15 on the Ipl1 sites drives excessive localization of Sli15 to the mitotic spindle in pre-anaphase cells. Consistent with these results, direct interaction of Sli15 with microtubules *in vitro* is greatly reduced either following phosphorylation by Ipl1 or when constitutive phosphorylation at the Ipl1-dependent phosphorylation sites is mimicked by aspartate or glutamate substitutions. Furthermore, we find that mimicking Ipl1 phosphorylation of Sli15 interferes with the ‘tension checkpoint’ – the CPC-dependent mechanism through which cells activate the spindle assembly checkpoint to delay anaphase in the absence of tension on kinetochore-microtubule attachments. Ipl1-dependent phosphorylation of Sli15 therefore inhibits its association with microtubules both *in vivo* and *in vitro* and may negatively regulate the tension checkpoint mechanism.

## Introduction

The chromosomal passenger complex (CPC) has emerged over the past decade as a critical and conserved regulator of eukaryotic cell division, with key roles in promoting chromosome bi-orientation, in the spindle assembly checkpoint, in spindle disassembly during anaphase and as a regulator of cytokinesis [1]. The CPC consists of a protein kinase (Aurora B/Ipl1) in association with three other proteins (INCENP/Sli15, Survivin/Bir1 and Borealin/Nbl1) and in metaphase is localized to centromeres, where it promotes chromosome bi-orientation on the mitotic spindle. This is thought to involve destabilization of incorrect microtubule-kinetochore connections through phosphorylation of Aurora B/Ipl1 targets at the kinetochore [2], [3], allowing turnover of connections until chromosomes achieve amphitelic attachment, which is most likely signaled by sister chromatids coming under tension from the kinetochore microtubules once they are correctly attached. Kinetochore localization of the CPC is promoted by Sgo1, which recognizes histone H2A following phosphorylation of its C-terminal tail by Bub1 [4], and by haspin kinase, which phosphorylates histone H3 on Thr-3 to create a binding site for Survivin [5], [6], [7]. However, in budding yeast the role of these mechanisms is less clear-cut [8], [9], [10] and direct interaction between Bir1 and the inner kinetochore protein Ndc10 may be utilized to target the CPC [11], [12].

During anaphase, the CPC undergoes dynamic relocalization to the spindle mid-zone [1], promoting spindle disassembly and cytokinesis. In yeast, the kinetochore protein Dam1 is a key Ipl1 substrate that mediates its role in chromosome bi-orientation [13], [14], while phosphorylation by Ipl1 of proteins such as Bim1 and She1 in anaphase are important for promoting spindle disassembly [15], [16]. Thus Ipl1-dependent phosphorylation of Bim1, a microtubule-stabilizing protein related to EB1 [17], promotes unloading of Bim1 from the spindle microtubules [15], [16], while phosphorylation of She1 appears to promote its activity as a spindle disassembly factor [15]. The dramatic relocalization of the CPC as cells enter anaphase is related to changes in the activity of cyclin-dependent kinases and their opposing phosphatases: INCENP/Sli15 is phosphorylated by cyclin-dependent kinase 1 (Cdk1) and this inhibits its interaction with the spindle [18], [19], while removal of Cdk1-dependent phosphorylation (in the case of Sli15 by Cdc14 phosphatase) is a key element driving interaction of the yeast CPC with the spindle mid-zone in anaphase [18]. Relocalization of the CPC to the spindle mid-zone is also important for preventing re-engagement of the spindle assembly checkpoint during anaphase when tension on microtubule-kinetochore attachments is reduced [20], [21] following loss of sister chromatid cohesion.

INCENP/Sli15 consists of three domains: the N-terminal region that mediates association with Bir1 and Nbl1, the central domain that binds microtubules and the C-terminal domain or IN-box that is involved in binding to and activating Aurora B/Ipl1 protein kinase [22], [23]. Sli15 is itself a substrate for phosphorylation by Ipl1, becoming phosphorylated by the kinase during *in vitro* protein kinase assays [23], while in higher eukaryotes phosphorylation of INCENP by Aurora B on a C-terminal Thr-Ser-Ser motif contributes to full activation of Aurora B [24], [25]. To examine whether Ipl1-dependent phosphorylation of Sli15 is relevant for CPC function or localization, we set out to identify the Ipl1-dependent phosphorylation sites and to examine the potential role that phosphorylation might play. A recent study in which predicted Ipl1 phosphorylation sites in Sli15 were changed to non-phosphorylatable alanine showed that in addition to Cdk1-dependent phosphorylation, Ipl1-dependent phosphorylation of Sli15 is also likely to regulate CPC interaction with the spindle and that in cooperation with Cdk1, Ipl1 phosphorylation of Sli15 helps to ensure appropriate microtubule dynamics at different stages in the cell cycle [26]. Here we report the identification of 14 sites in Sli15 that are phosphorylated directly by Ipl1 and confirm that Ipl1-dependent phosphorylation of Sli15 regulates CPC association with spindle microtubules. By mutating *SLI15* to encode a protein in which constitutive phosphorylation of Sli15 is mimicked, we demonstrate that phosphorylation *in vivo* is likely to limit the interaction of the CPC with the spindle, and show that phosphorylation of Sli15 or the acidic substitutions that mimic constitutive phosphorylation block the direct binding of Sli15 to microtubules in a novel *in vitro* binding assay. Furthermore, we find that mimicking constitutive phosphorylation of Sli15 on its Ipl1 phosphorylation sites interferes with the cell's ability to mount a spindle assembly checkpoint response specifically to reduced tension on sister kinetochores (the ‘tension checkpoint’). Thus in addition to affecting the spindle association of the CPC, Ipl1-dependent phosphorylation of Sli15 may act as a negative regulator of the tension checkpoint response.

## Materials and Methods

### Yeast strains and general methods

Basic yeast methods, growth media, and routine recombinant DNA methodology were performed as previously described [27], [28]. All yeast strains used in this study (Table 1) are derivatives of W303-1a [29] and have the following markers unless indicated to the contrary: *ade2-1 his3-11*, *15 leu2-3*, *112 trp1-1 ura3-1 can1-100 ssd1-d2* Gal^+^. Plasmids used or generated in this work are summarized in Table 2. Relative growth, temperature sensitivity and benomyl sensitivity were assessed by adjusting overnight cultures of strains to OD_600_ 0.1 and then spotting 5 µl of undiluted and 10-fold serial dilutions onto YPAD agar plates or plates containing 10, 11 or 12.5 µg/ml benomyl, followed by growth for 2 days at the indicated temperatures. Chromosome loss was examined by monitoring the loss of a chromosome III fragment (CFIII) carrying *URA3* and *SUP11* [30] in a colony-sectoring assay, where the loss of CFIII (and hence *SUP11*) leads to a red colony color due to the failure to suppress the *ade2-1* mutation.

**Table 1:** Yeast Strains.

Strains^a^	Genotype	Source
K699	*MAT*a	Kim Nasmyth
SJC591	*MAT*a *sli15Δ::KanMX6 his3*::*SLI15-S20A-EGFP*::*HIS3 trp1::pTEF1-mCherry-TUB1*::*TRP1 cdc20*::*pMET-CDC20*::*TRP1*	This study
SJC594	*MAT*a *ipl1(S50A,S76A)::HIS3MX6 sli15Δ::KANMX6 his3::sli15(S335A)::HIS3*	This study
SJC597	*MAT*a *ipl1(S50A,S76A)::HIS3MX6 sli15Δ::KANMX6 his3::sli15(20A)::HIS3*	This study
SJC600	*MAT*a *ipl1(S50E,S76E)::HIS3MX6 sli15Δ::KANMX6 his3::sli15(S335D)::HIS3*	This study
SJC603	*MAT*a *ipl1(S50E,S76E)::HIS3MX6 sli15Δ::KANMX6 his3::sli15(20D)::HIS3*	This study
SJC641	*MAT*a *ipl1(S50A,S76A)::HIS3MX6 sli15Δ::KANMX6 his3::SLI15::HIS3*	This study
SJC644	*MAT*a *ipl1(S50E,S76E)::HIS3MX6 sli15Δ::KANMX6 his3::SLI15::HIS3*	This study
SJC649	*ipl1(S50A,S76A)::HIS3MX6 sli15Δ::KANMX6 his3::sli15(20A,S335A)::HIS3*	This study
SJC655	*ipl1(S50E,S76E)::HIS3MX6 sli15Δ::KANMX6 his3::sli15(20D,S335D)::HIS3*	This study
VMY30	*MAT*a *sli15Δ::KANMX6 his3*::*SLI15*::*HIS3*	This study
VMY92	*MAT*a *CFIII (CEN3.L.YPH278) URA3 SUP11 sli15Δ::KANMX6 his3*::*SLI15*::*HIS3*	This study
VMY148	*MAT*a *sli15Δ::KANMX6 his3*::*SLI15-S20A*::*HIS3*	This study
VMY157	*MAT*a *CFIII (CEN3.L.YPH278) URA3 SUP11 sli15Δ::KANMX6 his3*::*SLI15-S20A*::*HIS3*	This study
VMY162	*MAT*a *sli15Δ::KANMX6 his3*::*SLI15-S20A*::*HIS3 pds1::PDS1*-myc_18_ *::LEU2*	This study
VMY166	*MAT*a *pds1::PDS1-myc_18_::LEU2 scc1Δ::TRP1 leu2::LEU2::pGAL-SCC1 sli15Δ::KANMX6 his3*::*SLI15-S20A*::*HIS3*	This study
VMY187	*MAT*a *sli15Δ::KANMX6 his3*::*SLI15-S20D*::*HIS3*	This study
VMY191	*MAT*a *sli15Δ::KANMX6 his3*::*SLI15-S20D*::*HIS3 pds1::PDS1*-myc_18_ *::LEU2*	This study
VMY194	*MAT*a *sli15Δ::KANMX6 his3*::*SLI15*::*HIS3 PDS1-myc_13_::NatRMX6*	This study
VMY222	*MAT*a *pds1::PDS1-myc_18_::LEU2 scc1Δ::TRP1 leu2::LEU2::pGAL-SCC1 sli15Δ::KANMX6 his3*::*SLI15*::*HIS3*	This study
VMY224	*MAT*a *CFIII (CEN3.L.YPH278) URA3 SUP11 sli15Δ::KANMX6 his3*::*SLI15-S20D*::*HIS3*	This study
VMY316	*MAT*a *sli15Δ::KANMX6 his3*::*SLI15*::*HIS3 cdc20*::*pMET-CDC20*::*TRP1 CEN5-tetO_336_::HIS3 leu2::tetR-GFP::LEU2 ura3::VENUS-TUB1::URA3*	This study
VMY318	*MAT*a *sli15Δ::KANMX6 his3*::*SLI15-S20A*::*HIS3 cdc20*::*pMET-CDC20*::*TRP1 CEN5-tetO_336_::HIS3 leu2::tetR-GFP::LEU2 ura3::VENUS-TUB1::URA3*	This study
VMY320	*MAT*a *sli15Δ::KANMX6 his3*::*SLI15-S20D*::*HIS3 cdc20*::*pMET-CDC20*::*TRP1 CEN5-tetO_336_::HIS3 leu2::tetR-GFP::LEU2 ura3::VENUS-TUB1::URA3*	This study
VMY356	*MAT*a *pds1::PDS1-myc_18_::LEU2 scc1Δ::TRP1 leu2::LEU2::pGAL-SCC1 sli15Δ::KANMX6 his3*::*SLI15-S20D*::*HIS3*	This study
VMY357	*MAT*a *sli15Δ::KANMX6 his3*::*SLI15-EGFP*::*HIS3 trp1::pTEF1-mCherry-TUB1*::*TRP1 cdc20*::*pMET-CDC20*::*TRP1*	This study
VMY361	*MAT*a *sli15Δ::KANMX6 his3*::*SLI15-S335D*::*HIS3*	This study
VMY363	*MAT*a *sli15Δ::KANMX6 his3*::*SLI15-S335A*::*HIS3*	This study
VMY365	*MAT*a *sli15Δ::KANMX6 his3*::*SLI15-S20A-S335A*::*HIS3*	This study
VMY367	*MAT*a *sli15Δ::KANMX6 his3*::*SLI15-S20D-S335D*::*HIS3*	This study
VMY375	*MAT*a *sli15Δ::KANMX6 his3*::*SLI15-S20D-EGFP*::*HIS3 trp1::pTEF1-mCherry-TUB1*::*TRP1 cdc20*::*pMET-CDC20*::*TRP1*	This study
VMYD16	*MAT*a/*MATα SLI15/sli15Δ::KANMX6*	This study

a All strains are in the W303 background: *ade2-1 his3-11,15 leu2-3,112 trp1-1 ura3-1 can1-100 ssd1-d2* Gal^+^.

**Table 2:** Plasmids.

Plasmid	Description	Source
pCJ145	pRS316-*SLI15*	Gislene Pereira
pMS53	pGEX-2T-*SLI15*	This study
pMS294	pGEX-2T-*DAM1*	Patrick Keating [34]
pMS295	pET28a-Ipl1- His_6_	This study
pMS84	pJ201-*sli15*-20D	This study
pMS85	pRS303-*sli15-S20D*	This study
pMS86	pRS303-*sli15-S20A*	This study
pMS218	pRS303-*SLI15*	This study
pMS176	pGEX-6P-GST-*SLI15*-His_6_	This study
pMS177	pGEX-6P-GST-*sli15-S20A*-His_6_	This study
pMS178	pGEX-6P-GST-*sli15-S20D*-His_6_	This study
pMS226	pRS303-*sli15-S335D*	This study
pMS227	pRS303-*sli15-S335A*	This study
pMS228	pRS303-*sli15-S20A*, *S335A*	This study
pMS229	pRS303-*sli15-S20D*, *S335D*	This study
pMS230	pRS303-*SLI15*-EGFP	This study
pMS231	pRS303-*sli15-S20A*-EGFP	This study
pMS232	pRS303-*sli15-S20D*-EGFP	This study

### *sli15* mutant alleles

For generating strains with mutant copies of *SLI15*, a 2.55 kb *Bam*HI*-Xho*I fragment carrying *SLI15* together with 300 bp of upstream and downstream flanking sequence was first excised from pCJ145 and inserted into pRS303. The *sli15-20A* plasmid was created from pRS303-*SLI15* by sequential site-directed mutagenesis using different primers on pRS303-*SLI15* with the QuikChange Site-Directed Mutagenesis Kit (Stratagene, La Jolla, CA) according to the manufacturer's instructions. The mutated plasmid was verified by DNA sequencing, linearized with *Bsi*WI and integrated in single copy at one of the *his3* loci in VMYD16 (*SLI15/sli15Δ::KanMX6*). Following sporulation and tetrad dissection, the required haploid *sli15Δ::KanMX6 his3::sli15-20A::HIS3* strains were generated. Control strains expressing wild-type *SLI15* were similarly generated using pRS303-*SLI15*. For creating the *sli15-20D* plasmid, a 1479 bp fragment of *SLI15* encoding 20 substitutions to glutamate or aspartate was synthesized (DNA 2.0, Menlo Park, California) and subcloned as an *Nco*I-*Mfe*I restriction fragment in place of the equivalent wild-type fragment in pRS303-*SLI15*, generating pRS303-*SLI15-20D*. pRS303-*SLI15-20D* was integrated in VMYD16 as above. Following tetrad dissection, high levels of spore inviability were seen that were rescued following transformation with pCJ145 (pRS316-*SLI15*). *sli15Δ::KanMX6 his3::sli15-20D::HIS3* strains were therefore generated by sporulation and tetrad dissection of the pCJ145-containing strain, followed by two rounds of selection on 5-fluoroorotic acid to evict the plasmid.

### Spindle assembly checkpoint analysis

Whole-cell extracts were made and immunoblotted as previously described [31]. Briefly, for synchronization of cells in G1, 1.25 µg/ml α-factor was used. To prevent cells from entering the next cell cycle after release from α-factor arrest, α-factor was added back (7.5 µg/ml) when small buds appeared in the majority (>80%) of cells. Nocodazole was used at 30 µg/ml. *pGAL-SCC1* shut-off was performed as previously described [31]. Cell cycle progression was studied by monitoring budding and Pds1 levels in strains expressing *PDS1-myc*_18_. For lysing cells sequential NaOH and trichloroacetic acid treatment was used [32]. Pds1-myc_18_ was detected by Western blotting with an anti-*myc* antibody (c-*myc* A-14; sc-789; Santa Cruz Biotechnology). Cdc28 was detected using an anti-Cdc28 antibody from Santa Cruz Biotechnology (sc-6709). GFP was detected with anti-GFP (11814460001; Roche).

### Microscopy

The time-lapse fluorescence microscopy of live cells for monitoring chromosome biorientation was performed as previously described [31]. Briefly, time-lapse images of cells immobilized on agarose pads released from G1 (α-factor) to metaphase arrest (Cdc20 depletion) were collected for 4 min using a DeltaVision RT microscope (Applied Precision), a UplanSApo 100× objective lens (numeric aperture 1.40; Olympus), SoftWoRx software (Applied Precision), and a CoolSnap HQ (Photometrics) charge-coupled device camera. Seven Z sections (0.3 µm apart) were acquired and subsequently deconvolved, projected as two-dimensional images, and analyzed with SoftWoRx software. VENUS and GFP fluorescent protein signals were visualized with a JP3 filter set, whereas for GFP and mCherry fluorescent proteins an ET filter set was used. Sister *CEN5* centromeres that remained unseparated at one end of the metaphase spindle were scored as mono-oriented, whereas sister *CEN5* centromeres that showed dynamic separation and reassociation were scored as bioriented. At least 100 cells were analyzed for all reported experiments. Quantification of Sli15-EGFP fluorescence was performed with Volocity software (PerkinElmer). Statistical analysis of chromosome biorientation data was carried out using Fisher's exact test. Statistical analysis of all other microscopy data made use of the Mann-Whitney *U* test. All *P* values are two tailed.

### Protein expression and purification

Recombinant GST-Ipl1, GST-Sli15, Ipl1-His_6_ and GST-Dam1 were expressed and purified essentially as described previously [33], [34]. To prepare wild-type GST-Sli15-His_6_, GST-Sli15-20A-His_6_ and GST-Sli15-20D-His_6_, *E. coli* BL21(DE3) cells transformed with the appropriate construct were induced with 100 µM IPTG at OD_600_ = 0.6 for 12–16 h at 22°C. Cells were harvested by centrifugation at 5000 rpm, 4°C. Bacterial pellets were washed once with chilled PBS and resuspended in Lysis Buffer (50 mM Bis-Tris propane pH 7.5, 300 mM KCl, 1% NP40, 1 mM EDTA, 1 mg/ml lysozyme, 10 µg/ml DNase, 5 mM DTT and Roche Complete Protease Inhibitor Cocktail). Sonicated lysates were cleared by centrifugation at 40,000 rpm for 30–45 min in a Beckman Ti45 rotor at 4°C. Supernatants were filtered with 0.2 µm filters and incubated with 1 ml of Glutathione Sepharose 4 Fast Flow (GE Healthcare) per liter of bacterial culture. Beads were prewashed with PBS (10 mM Na_2_HPO_4_, 1.8 mM KH_2_PO_4_, 137 mM NaCl, 2.7 mM KCl) and equilibrated with Lysis Buffer. After incubation for 1 h at 4°C, beads were transferred to a 10 ml Econo-Pac column (BioRad), washed with five column volumes of Lysis Buffer containing 1 M KCl prior to elution in 1 ml fractions with 40 mM reduced glutathione (Sigma-Aldrich), 100 mM Tris-HCl (pH 8.0), 300 mM NaCl, 10%(v/v) glycerol. Elution fractions were assessed by SDS-PAGE and appropriate fractions pooled and dialyzed in 2 L Buffer I (50 mM Bis-Tris propane pH 7.5, 300 mM KCl, 10%(v/v) glycerol, 5 mM β-mercaptoethanol, 30 mM imidazole) overnight. Dialyzed protein solutions were diluted to 10 ml in Buffer I, and incubated with 200 µl of equilibrated Ni-NTA slurry (Qiagen) for 2 h at 4°C with mixing by rotation. The beads were transferred to a 10 ml Econo-Pac column (BioRad), washed with three column volumes Buffer I containing 1 M KCl, followed by a two column volume wash in Buffer I. The bound proteins were eluted by the addition of 0.2 ml Buffer I containing 250 mM imidazole (Sigma-Aldrich). Eluted fractions were assessed by SDS-PAGE and appropriate fractions dialyzed in 2 L 50 mM Bis-Tris propane (pH 6.8), 150 mM KCl, 100 mM imidazole, 20%(v/v) glycerol and 5 mM β-mercaptoethanol. Aliquots of 200 µl were snap-frozen and stored at −80°C.

### Phosphorylation site mapping

GST-Ipl1 kinase (∼2 µg) and GST-Sli15 (∼5 µg) prepared as described previously [33] were incubated for 30 min at 30°C in buffer containing 50 mM Tris-HCl (pH 7.5), 0.1% 2-mercaptoethanol, 0.1 mM EGTA, 10 mM MgCl_2_ and 100 µM [γ-^32^P]ATP (5000 cpm/pmol) in a total reaction volume of 200 µl. The reaction was stopped by adding SDS, dithiothreitol (DTT) and Sample Buffer (Invitrogen) to give final concentrations of 1% SDS, 10 mM DTT and 1× Sample Buffer, then the samples were heated at 70°C for 5 min and separated by SDS-PAGE on 10% polyacrylamide gels, stained with Colloidal Coomassie (Invitrogen) and phosphoprotein localized by autoradiography. The ^32^P labeled proteins were excised and the gel pieces were digested with either endoproteinase Lys-C or with trypsin, then peptides separated by HPLC and analyzed by MALDI-TOF-TOF mass spectrometry using a Biosystems 4700 Proteomics Analyzer and Edman degradation as described previously [35].

### Protein kinase assays

Protein kinase assays using recombinant GST-Sli15-His_6_ (0.06 µg) and Ipl1-His_6_ (0.3 µg) were carried out in 20 µl reactions with [γ-^32^P]ATP (>800 cpm/pmol) essentially as previously described [33], [34], using either 5 µg GST-Dam1, 1 µg histone H3.3 (kind gift from Professor Tom Owen-Hughes University of Dundee, UK) or 1 µg Myelin Basic Protein (MBP) as substrates. For two-step reactions involving a pre-incubation step GST-Ipl1 (0.6 µg/20 µl) and GST-Sli15 (0.5 µg/20 µl) were used and reactions were scaled up appropriately to allow for analysis of multiple samples. Microcystin (a kind gift from Professor C. MacKintosh, University of Dundee) was used at 1 µM and recombinant human protein phosphatase 1 (PP1γ) at 0.05 µg per assay. Phosphorylated proteins were separated by SDS-PAGE, detected by autoradiography and quantitated by liquid scintillation counting after excising the radiolabelled bands.

### Measurement of Sli15 and microtubule binding affinity using Biolayer Interferometry

Tubulin (250 µg) purified from bovine brain (TL238A; Cytoskeleton, Inc.) was mixed with 20 µg biotin labeled tubulin (T333P; Cytoskeleton, Inc.) in ice-cold PME buffer (80 mM Pipes-KOH pH 6.8, 1 mM MgCl_2_, 1 mM EGTA) containing 1 mM GTP (BST06-001; Cytoskeleon, Inc) and 1 mM DTT was thawed and centrifuged to remove insoluble protein for 5 min at 42,000 rpm using a Beckman TLA100 rotor at 4°C. To assemble microtubules, the reaction was incubated at 37°C for 30 min while taxol (TXD01; Cytoskeleton, Inc.) was added in a stepwise manner to a final concentration of 20 µM to stabilize microtubules. Microtubules were then centrifuged at 50,000 rpm for 5 min at 30°C and the pellet was resuspended in warm PME buffer containing 1 mM GTP, 1 mM DTT and 20 µM taxol to a final concentration of 5 µM polymerized tubulin.

Binding assays were carried out by biolayer interferometry at 25°C in solid black 96-well plates (Greiner Bio-One) using an Octet Red384 (ForteBio, Inc.). Streptavidin-coated biosensors (SA biosensors, ForteBio, Inc.) were equilibrated in Buffer A (80 mM Pipes-KOH pH 6.8, 1 mM MgCl_2_, 1 mM EGTA, 20 mM taxol), then loaded with 5 µM biotinylated polymerized tubulin (MTs). Unbound MTs were removed in a 5 min wash with Buffer A, followed by a subsequent wash with Buffer A containing 10 µM biocytin (Tocris) to block free streptavidin sites. Biocytin was removed in a 5 min wash step with Buffer A and the system was equilibrated for 5 min with Buffer B (50 mM Bis-Tris propane pH 6.8, 150 mM KCl, 20%(v/v) glycerol, 5 mM β-mercaptoethanol, 100 mM imidazole, 1 mg/ml BSA, 20 mM taxol). A parallel set of SA biosensors was blocked with biocytin to act as a reference surface to correct for non-specific binding of protein to the biosensors. Serial dilutions of GST-Sli15-His_6_, GST-Sli15-20A-His_6_, GST-Sli15-20D-His_6_ and GST control were allowed to associate in Buffer B for 5 min and dissociation from MTs was monitored for 10 min. Data were processed using ForteBio Data Analysis Software 7.0 to determine binding. In the kinase assay reaction, Ipl1-His_6_ was incubated with GST-Sli15 in the presence and absence of unlabeled 10 µM ATP for 30 min at 30°C as previously described [33] and subsequently association and dissociation from microtubules was monitored as described above.

## Results

### Identification of Ipl1 phosphorylation sites in Sli15

**Figure 1:**
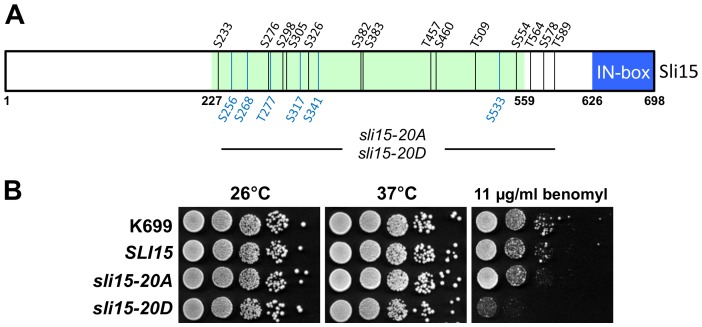
Characterization of the *sli15-20A* and *sli15-20D* alleles. (**A**) Schematic representation of Sli15 showing the 14 phosphorylation sites mapped *in vitro* (black) and the six additional sites discussed in the text (blue) that were mutated in *sli15-20A* and *sli15-20D*. The microtubule-binding region (residues 227–559) [23] is shaded green and the conserved IN box (residues 626–698) is shaded blue. (**B**) Equivalent 10-fold dilutions of a wild-type strain (K699) and of *sli15Δ::KanMX6* strains with either wild-type *SLI15* (VMY30), *sli15-20A* (VMY148) or *sli15-20D* (VMY187) integrated at the *his3* locus were spotted onto YPAD agar in the presence of absence of benomyl at 11 µg/ml and grown for two days at 26°C or 37°C as indicated.

Since Sli15 rapidly becomes phosphorylated by Ipl1 during *in vitro* protein kinase assays [23], we undertook the identification of those sites in Sli15 that are directly phosphorylated by Ipl1 so that their potential role in CPC function could be tested. Following an *in vitro* phosphorylation reaction, radiolabelled phosphopeptides were separated by HPLC, identified by mass spectrometry and the phosphorylation sites in each established following Edman degradation. In this way fourteen phosphorylation sites were found, of which all but one were identified with high confidence (Table 3). Except for the three sites closest to the C-terminus of Sli15, all of these phosphorylation sites are located within the central domain of Sli15 (Figure 1A) that is involved in binding to microtubules [23] and is also required for chromosome biorientation on the mitotic spindle [36]. Sli15 contains 17 matches to the consensus motif [KR]-X-[ST]-[ILVST] proposed for Aurora B/Ipl1 phosphorylation by Cheeseman *et al.* [13]. Ten of our mapped phosphorylation sites match this consensus (Table 4), while a further two of our mapped sites have asparagine in the +1 position, which was found to be the third most favored +1 residue in a recent large study of Aurora B-dependent phosphorylation [37]. All 14 sites have a basic residue in the –2 position that is a hallmark of Aurora B/Ipl1 phosphorylation sites (Table 4). Phosphopeptides corresponding to six of our mapped sites have also been identified *in vivo* in large-scale phosphoproteomics studies (Table 4); our work therefore strengthens the link between Ipl1 and these *in vivo* sites by demonstrating that Ipl1 is capable of phosphorylating them.

**Table 3:** Identification of Ipl1 phosphorylation sites on Sli15 following *in vitro* phosphorylation.

Enzyme	Mode a	m/z^b^ observed	m/z^b^ theoretical	Location in Sli15	Sequence^c^	Number of phosphates	Phosphorylated residues
Lys-C	R	2870.37	2870.50	218–242	(K)VKPPPNSGIARSQRRsNMFVPLPNK	1	Ser-233
Lys-C	R	2887.09	2888.28	218–242	(K)VKPPPNSGIARSQRRsNmFVPLPNK	1	Ser-233
Lys-C	L	3771.93	3769.96	276–310	(K)sTINSPAIRAVENSDTAGSTKAsSVFDRLSSIPTK	2	Ser-276, Ser-298
trypsin	L	2828.54	2829.88	285–310	(R)AVENSDTAGSTKAsSVFDRLsSIPTK	2	Ser-298, Ser-305
trypsin	R	2160.96	2160.96	319–338	(R)GNVGHKYsSSSIDLTGSPmK	1	Ser-326
Lys-C	R	2501.10	2501.19	316–338	(K)ISRGNVGHKYsSSSIDLTGSPMK	1	Ser-326
Lys-C	R	2517.02	2517.18	316–338	(K)ISRGNVGHKYsSSSIDLTGSPmK	1	Ser-326
Lys-C	R	1594.72	1594.71	378–389	(K)NSRKssIPRFDK	2	Ser-382, Ser-383
Lys-C	R	2698.98	2699.27	444–465	(K)NYYQSPVRGYLRPtKAsISPNK	2	Thr-457, Ser-460
trypsin	R	1611.88	1611.83	452–465	(R)GYLRPTKAsISPNK	1	Ser-460
Lys-C	L	2549.92	2550.79	505–525	(K)NYRLtNLQLLPPAEAERDDLK	1	Thr-509
Lys-C	R	2549.15	2549.28	505–525	(K)NYRLtNLQLLPPAEAERDDLK	1	Thr-509
trypsin	R	1336.65	1336.61	552–561	(K)RMsHLEQDLK	1	Ser-554
trypsin	R	1352.64	1352.60	552–561	(K)RmsHLEQDLK	1	Ser-554
Lys-C	R	1336.60	1336.61	552–561	(K)RMsHLEQDLK	1	Ser-554
Lys-C	R	1352.58	1352.60	552–561	(K)RmsHLEQDLK	1	Ser-554
Lys-C	R	1297.54	1297.55	562–571	(K)KQtSFSNDYK	1	Thr-564
Lys-C	L	2643.22	2642.78	572–592	(K)DIRLKEsLAPFDNHVRDtINK	2	Ser-578, Thr-589

a L, linear mode; R, reflector mode.

b m/z values are average [M+H]^+^ for linear mode and monoisotopic [M+H]^+^ for reflector mode.

c **s**, phosphoserine; **t**, phosphothreonine; **m**, oxidized methionine; (K), (R), residue preceding trypsin/Lys-C cleavage site.

**Table 4:** Comparison of *in vivo* and *in vitro* Ipl1 phosphorylation sites in Sli15.

Observed a ^,^ b	Mutated in *sli15-20A* and *sli15-20D* b	Mutated in *sli15-17A* c	Detected *in vivo* d	Sequence context^e^
		137	-	RF**S**I
		177	177	RE**S**S
233	233		233	RR**S**N
	256		-	KS**S**G
	268		268^f^	KE**S**P
276	276	276	276	KK**S**T
	277	277	-	KS**T**I
298	298	298	-	KA**S**S
305	305	305	-	RL**S**S
	317		-	KI**S**R
326	326	326	-	KY**S**S
	341		-	KV**S**Q
382	382	382	382	RK**S**S
383	383	383	383	KS**S**I
		391	-	KT**S**L
		395	-	KL**T**T
		413	-	KH**S**S
		432	432	KI**S**V
457	457		-	RP**T**K
460	460	460	-	KA**S**I
			489^f^	KL**S**P
509	509		-	RL**T**N
	533		533	RL**S**G
554	554		554	RM**S**H
564	564	564	-	KQ**T**S
578	578	578	578	KE**S**L
589	589	589	-	RD**T**I

a See Table 3 for phosphorylation site mapping data.

b This study.

c Nakajima *et* al. [26]; these sites represent all occurrences of the motif [KR]-X-[ST]-[ILVST] in Sli15.

d All verified *in vivo* phosphorylation sites present in the PhosphoGrid [50], [51] and Phosphopep databases [52] that have either K or R in the −2 position as required for potential Aurora B/Ipl1 phosphorylation, but without restricting the identity +1 residue.

e Phosphorylated residue is shown in bold.

f Possible *in vivo* target of a proline-directed protein kinase such as Slt2 or Cdc28 rather than Ipl1.

To investigate the possible functional significance of Sli15 phosphorylation by Ipl1 we mutated all 14 mapped sites to non-phosphorylatable alanine residues, together with six additional sites close to mapped sites that also conformed to the Ipl1 consensus motif of Cheeseman *et al.* [13], two of which are known to be phosphorylated *in vivo* (Table 4). This allele (encoding Sli15 with 20 alanine substitutions) was termed *sli15-20A*. A corresponding phosphomimic allele (*sli15-20D*) was also generated in which the serine and threonine residues at these twenty sites were replaced with aspartate or glutamate respectively. Detection of the wild-type and mutant versions of Sli15 following EGFP-tagging indicated that all three proteins were present at similar levels in asynchronously growing cells, and also in metaphase-arrested or anaphase cells, confirming that neither the *sli15-20A* nor *sli15-20D* alleles conferred a significant change in the level of Sli15 in comparison to the wild-type protein (data not shown). Despite the multiple amino acid replacements, yeast cells relying on either *sli15-20A* or *sli15-20D* as their sole source of Sli15 grew normally at 26°C and 37°C, indicating that both mutant proteins can provide sufficient Sli15 function to support normal rates of proliferation (Figure 1B). However, in contrast to *sli15-20A*, the *sli15-20D* allele conferred considerable hypersensitivity to the microtubule depolymerizing drug benomyl (Figure 1B).

### Sli15 phosphorylation is not required for Ipl1 kinase activation

**Figure 2:**
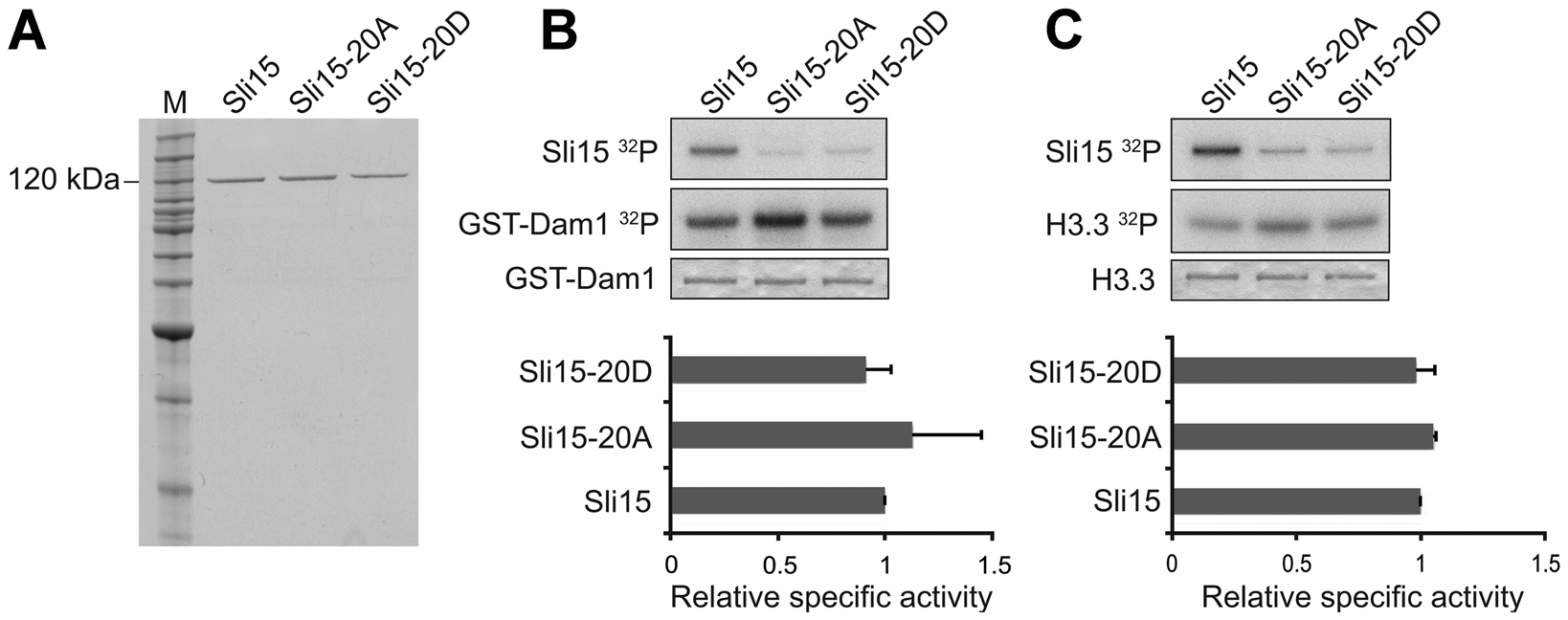
*In vitro* Ipl1-dependent phosphorylation promoted by wild-type and mutant versions of Sli15. (**A**) Coomassie-stained gel showing recombinant GST-Sli15-His_6_ preparations used for *in vitro* phosphorylation and microtubule binding. (**B**) Phosphorylation of GST-Dam1 by Ipl1 in the absence or presence of wild-type (Sli15) or mutant forms (Sli15-20A, Sli15-20D) of GST-Sli15-His_6_. Top panel, GST-Sli15-His_6_ phosphorylation (^32^P incorporation from [γ-^32^P]ATP); middle panel, GST-Dam1 phosphorylation (^32^P incorporation from [γ-^32^P]ATP); lower panel, GST-Dam1 detected by Coomassie staining. (**C**) Phosphorylation of H3.3 by Ipl1 in the absence or presence of wild-type or mutant forms of GST-Sli15-His_6_. Top panel, GST-Sli15-His_6_ phosphorylation (^32^P incorporation from [γ-^32^P]ATP); middle panel, H3.3 phosphorylation (^32^P incorporation from [γ-^32^P]ATP); lower panel, H3.3 detected by Coomassie staining. The histograms show mean relative specific activity (n = 3) of H3.3 (B) and GST-Dam1 (C) phosphorylation by Ipl1 promoted by GST-Sli15-20A-His_6_ or GST-Sli15-20D-His_6_ in comparison with GST-Sli15-His_6_ (arbitrarily set to 1.0).

We next compared the ability of Sli15, Sli15-20A and Sli15-20D to promote the protein kinase activity of Ipl1 in an *in vitro* assay. Since activation is primarily a function of the conserved C-terminal IN-box [23] whereas the phosphorylation sites are all located upstream of this region, it was not anticipated that mutation of the phosphorylation sites would affect activation of Ipl1. Figure 2 shows that wild-type Sli15, Sli15-20A and Sli15-20D were each able to promote similar levels of phosphorylation by Ipl1 of either Dam1 (Figure 2B) or histone H3 (Figure 2C), indicating that phosphorylation of Sli15 by Ipl1 is not intrinsically required for full activation of its kinase activity and consistent with the established role of the IN-box. Since the central domain of Sli15 has been shown to interact with Dam1 as well as with microtubules [23], these results also indicate that phosphorylation of Sli15 by Ipl1 is unlikely to affect the interaction between the yeast CPC and its critical substrate at the kinetochore, since all three GST-Sli15 fusions promoted Dam1 phosphorylation with similar efficiency. Both Sli15-20A and Sli15-20D showed a major reduction in phosphorylation in comparison with wild-type GST-Sli15 in these assays, consistent with the notion that the 20 sites that we have mutated encompass most if not all major sites of Ipl1 phosphorylation within Sli15.

**Figure 3:**
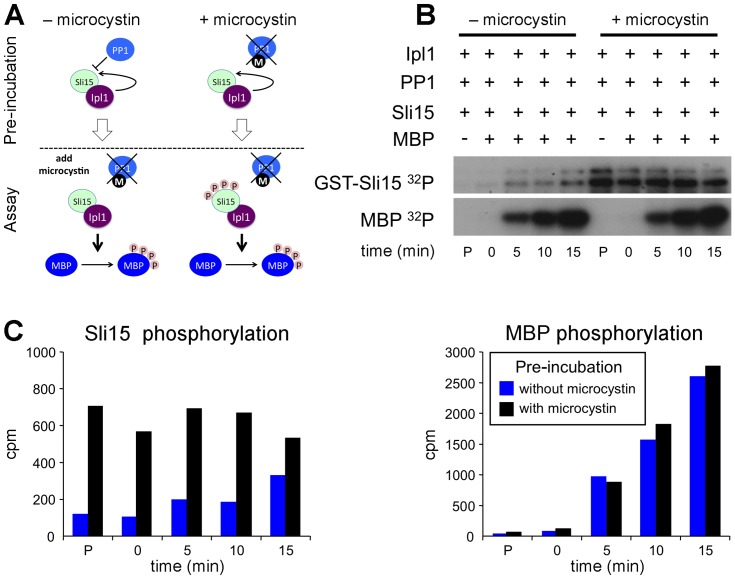
Ipl1 activation by Sli15 is independent of Sli15 phosphorylation. GST-Ipl1 (Ipl1), GST-Sli15 (Sli15), recombinant PP1γ and [γ-^32^P]ATP were pre-incubated for 20 min, a time that allowed maximal phosphorylation of Sli15 in the absence of PP1γ, in either the presence or absence of the PP1 inhibitor microcystin. A sample (P) was taken, then microcystin was added to the reaction that lacked the inhibitor and myelin basic protein (MBP) added to both reactions, taking further samples over a 15-minute time course. All samples were separated by SDS-PAGE and both MBP and Sli15 phosphorylation monitored by autoradiography. (**A**) Schematic showing the experimental design. (**B**) Autoradiograph. (**C**) Quantitation of Sli15 and MBP bands shown in (B) by liquid scintillation counting after excising the labelled bands.

To confirm that Sli15 phosphorylation by Ipl1 is not involved in full kinase activation, we compared the ability of Sli15 that was either phosphorylated or not phosphorylated on the Ipl1-dependent sites to promote Ipl1-dependent phosphorylation of an exogenous substrate. To generate these two conditions the scheme shown in Figure 3A was used in which Ipl1, Sli15 and protein phosphatase 1 (PP1) were first pre-incubated with radiolabelled ATP either in the presence or absence of the PP1 inhibitor microcystin. In its presence, Sli15 became phosphorylated as expected, whereas in the absence of microcystin the active PP1 opposed Sli15 phosphorylation, as indicated by lack of ^32^P incorporation into Sli15 (Figure 3; lanes labelled P). At the end of the pre-incubation, microcystin was added to the reaction without inhibitor and then both reactions were supplemented with myelin basic protein (MBP), which is a good substrate for Ipl1-Sli15. MBP phosphorylation in both reactions progressed at essentially identical rates, while the stable level of ^32^P incorporation into Sli15 following pre-incubation with inhibitor indicates that it had become maximally phosphorylated during the pre-incubation step (Figure 3). Thus the initial phosphorylation state of Sli15 had no significant influence on its ability to activate Ipl1 and is therefore consistent with the ability of both Sli15-20A and Sli15-20D to activate Ipl1 to a similar extent as wild-type Sli15 *in vitro* (Figure 2).

### Mutation of Ipl1 phosphorylation sites on Sli15 causes elevated levels of chromosome loss but does not compromise chromosome bi-orientation

**Figure 4:**
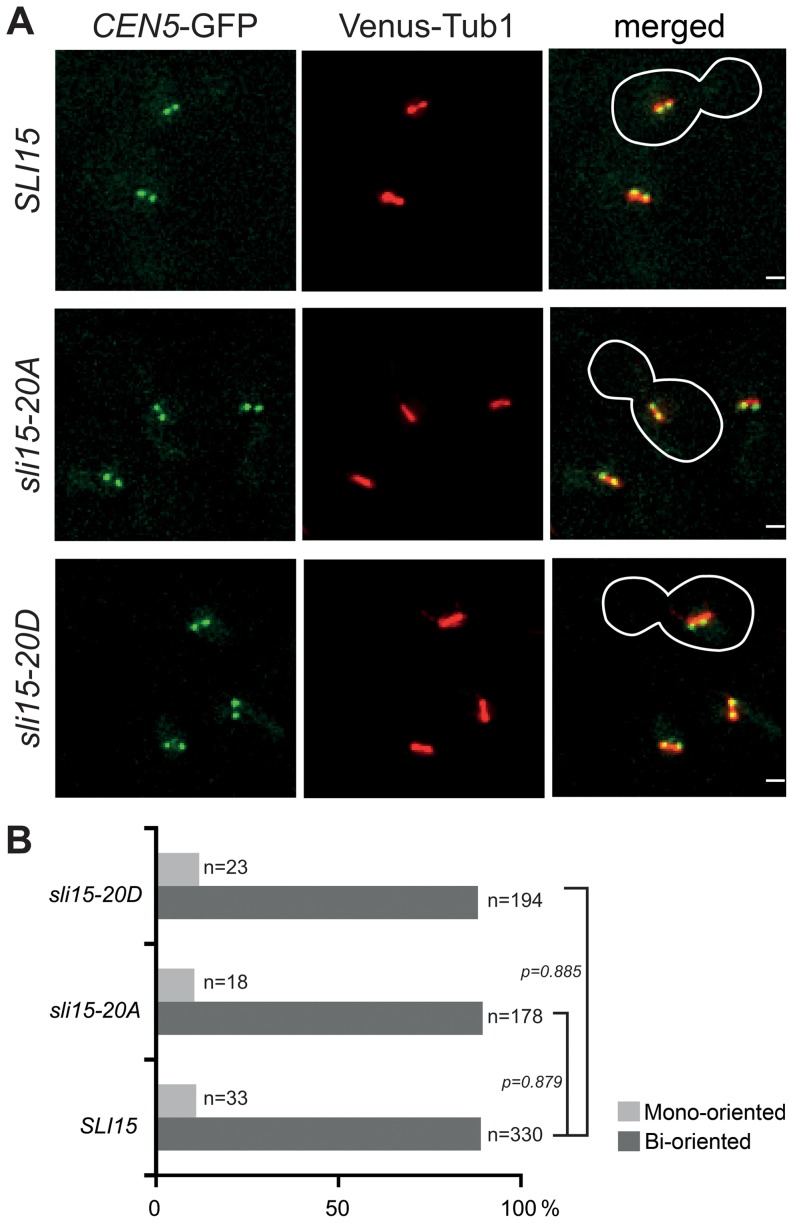
Ipl1-dependent Sli15 phosphorylation is dispensable for chromosome bi-orientation. Wild type *SLI15* (VMY316), *sli15-20A* (VMY318) and *sli15-20D* (VMY320) cells containing *CEN5*-(*tetO)_336_, tetR*-GFP, Venus-*TUB1* and p*MET3-CDC20* were arrested in G1 with α-factor at 26°C and then released to a metaphase block in rich medium (containing 2 mM methionine to deplete Cdc20) for 2.5 h. (**A**) Representative stills from time-lapse images of live cells. Bi-oriented chromosomes show dynamic splitting and reassociation of sister *CEN5s*. Green, *CEN5* labeled with *tetR*-GFP; red, Venus-tubulin. (**B**) Quantification of chromosome bi-orientation in metaphase-arrested cells from multiple time-lapse fields (n = number of cells scored in each category).

To investigate whether Ipl1-dependent phosphorylation of Sli15 plays a role in chromosome stability, chromosome loss rates were measured using a colony-sectoring assay in wild-type, *sli15-20A* and *sli15-20D* strains. As shown in Table 5, both *sli15-20A* and *sli15-20D* led to an approximately 10-fold increase in chromosome loss rate. Thus either blocking or constitutively mimicking phosphorylation reduced the fidelity of chromosome transmission, indicating that both mutant proteins are functionally compromised in some way and suggesting that phosphorylation of Sli15 by Ipl1 plays a role in ensuring accurate chromosome segregation. A key role of the CPC in yeast and other eukaryotes is in the establishment of chromosome bi-orientation, and both *ipl1* and *sli15* temperature-sensitive mutants show high levels of mono-oriented chromosomes at their restrictive temperatures [2]. Thus elevated chromosome loss in the *sli15* mutants could result from failure of the CPC to promote chromosome biorientation. We therefore generated strains containing the *sli15-20A* or *sli15-20D* alleles in which we could monitor chromosome biorientation through the behavior of GFP-labelled sister *CEN5*s in cells arrested in metaphase by Cdc20 depletion. Bioriented chromosomes show dynamic splitting and reassociation of GFP-labelled sister centromeres in metaphase-arrested cells, whereas mono-oriented chromosomes show a single, unresolved GFP locus at one end of the metaphase spindle [31]. As shown in Figure 4, chromosome biorientation was unaffected by either *sli15-20A* or *sli15-20D*, occurring with the same efficiency as in the *SLI15* wild-type control strain. Thus inefficient chromosome biorientation cannot account for the elevated rate of chromosome loss conferred by either the *sli15-20A* or the *sli15-20D* allele.

**Table 5:** *sli15-20A* and *sli15-20D* show elevated rates of chromosome loss.

Strain	Red colonies	Half-sectored colonies	Total colonies	Chromosome loss rate per cell division (× 10^3^)^a^	Fold increase
wt	17	6	4160	1.45	-
*sli15-20A*	48	55	4140	13.4	9.3
*sli15-20D*	114	75	5088	15.1	10.4

a Calculated as [half-sectored]÷([total]−[red]).

### Mimicking constitutive Ipl1 phosphorylation of Sli15 compromises the spindle assembly checkpoint response to kinetochores that are not under tension

**Figure 5:**
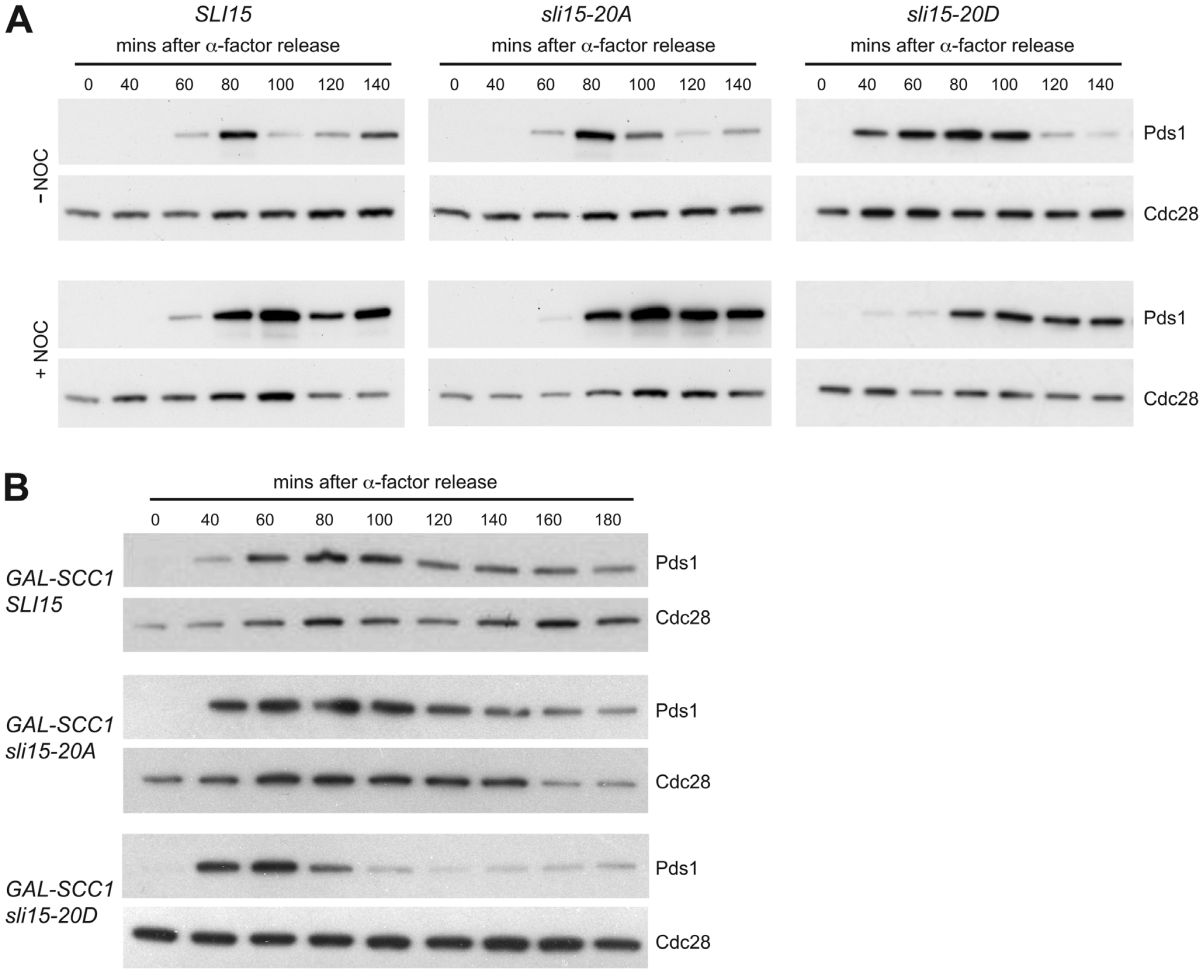
Mimicking constitutive Ipl1-dependent phosphorylation of Sli15 interferes with the checkpoint response to reduced cohesion. (A) Wild-type *SLI15* (VMY194), *sli15-20A* (VMY162), and *sli15-20D* (VMY191) strains expressing Pds1-*myc*_18_ were arrested in G_1_ with α-factor and synchronously released into YPD medium in the presence (+NOC) or absence (−NOC) of 30 µg/ml nocodazole. Samples were collected at the indicated times. Levels of Pds1-myc_18_ (Pds1) and Cdc28 (loading control) were monitored by immunoblotting using anti-*myc* and anti-Cdc28 antibodies, respectively. (B) Wild-type p*GAL-SCC1 SLI15* (VMY222), p*GAL-SCC1 sli15-20A* (VMY166) and p*GAL-SCC1 sli15-20D* (VMY356) cells expressing Pds1-*myc*_18_ were arrested with α-factor for 2 h in medium containing galactose and then released in medium containing glucose to repress p*GAL-SCC1*. Pds1 and Cdc28 were monitored as described for panel A.

The spindle assembly checkpoint monitors kinetochore-microtubule interactions during chromosome alignment on the mitotic spindle, delaying the onset of anaphase when chromosomes are either unattached or mono-oriented and therefore not under tension from the spindle microtubules [38]. Although Ipl1 is not required for activation of the spindle assembly checkpoint in response to microtubule depolymerisation, it is specifically needed for cells to delay anaphase onset when chromosomes are incorrectly attached to microtubules, leading to a lack of tension on microtubule-kinetochore attachments [39], [40]. Ipl1's role in this ‘tension checkpoint’ may be either through generating unattached kinetochores in the process of promoting chromosome biorientation [41], or alternatively by a more direct role in the checkpoint mechanism [33], [42]. Since mutations that interfere with the spindle assembly checkpoint mechanism confer hypersensitivity to microtubule depolymerizing agents such as nocodazole, we considered it possible that the *sli15-20D* allele might confer a checkpoint defect. However, when *sli15-20A*, *sli15-20D* and control strains were synchronized in G1 with α-factor and then released in the presence of nocodazole, all three showed a robust delay to anaphase in comparison to untreated cells as indicated by persistence of the anaphase inhibitor Pds1 (Figure 5A), whose destruction marks anaphase onset [43].

To examine the checkpoint response of the *sli15* mutants when sister kinetochores are not under tension, strains in which the cohesin subunit Scc1 was expressed from the *GAL* promoter were synchronized in G1 and then released in the presence of glucose to repress Scc1 expression. In the ensuing S-phase, sister chromatid cohesion is not established, leading to failure of kinetochore-microtubule attachments to come under tension from spindle microtubules [44]. Figure 5B shows that while the kinetics of Pds1 destruction were similar in the wild-type and *sli15-20A* strains, Pds1 was destroyed at least 40 minutes earlier in the *sli15-20D* strain, indicating that in comparison with the other two strains it was unable to delay anaphase noticeably when sister kinetochores are not under tension. Thus in contrast to alanine substitution of the six Cdk sites in Sli15 that prevents engagement of the spindle assembly checkpoint in response to reduced sister chromatid cohesion [20], preventing phosphorylation of Sli15 by Ipl1 does not appear to compromise the tension checkpoint response. In contrast, though, the tension checkpoint is rendered ineffective by mimicking constitutive Sli15 phosphorylation by Ipl1.

### Sli15-20A and Sli15-20D show altered interaction with microtubules *in vivo* and *in vitro*

**Figure 6:**
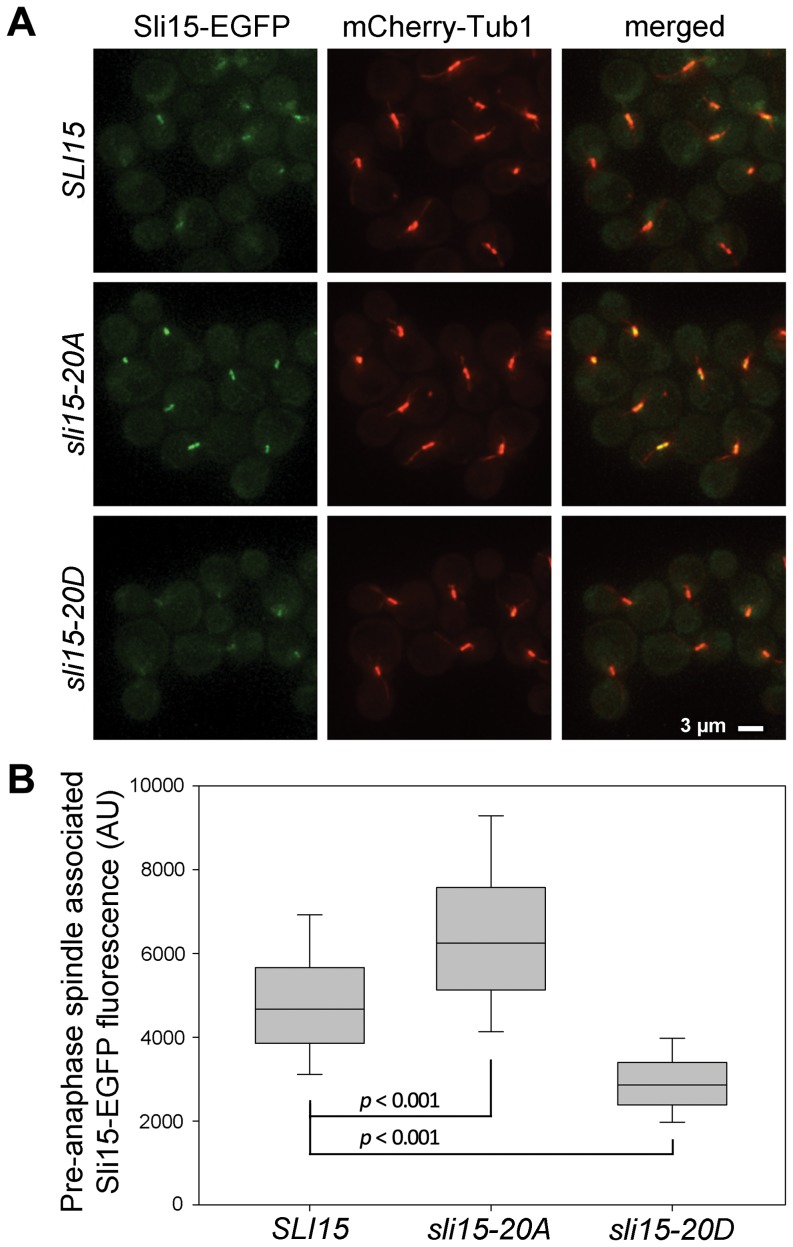
Localization of Sli15, Sli15-20A and Sli15-20D in metaphase-arrested cells. VMY357 (*SLI15*-EGFP), SJC591 (*sli15-20A*) and VMY375 (*sli15-20D*) cells containing mCherry-*TUB1* and p*MET3-CDC20* were arrested in G1 with α-factor at 26°C and then released to a metaphase block in rich medium for 2.5 h. (**A**) Representative images of live cells arrested in metaphase. (**B**) Quantification of spindle associated Sli15-EGFP fluorescence in cells from multiple fields imaged 2.5–3.5 h following α-factor release. The top and bottom of the boxes indicate the 75^th^ and 25^th^ percentiles and the whiskers indicate the 10^th^ and 90^th^ percentiles. Significance levels were determined using the Mann-Whitney *U* test of the null hypothesis that the medians were identical. *SLI15* vs. *sli15-20A*: Mann-Whitney *U* = 36165, n*_SLI15_* = 378, n*_sli15-20A_* = 387, *p*<0.001, two tailed. *sli15-20D* vs. *SLI15*: Mann-Whitney *U* = 15992, n*_sli15-20D_* = 381, n*_SLI15_* = 378, *p*<0.001, two tailed. Data shown are representative of three separate experiments. AU, arbitrary units of fluorescence.

Since the Ipl1 phosphorylation sites in Sli15 are largely located within the region known to interact with microtubules [23], we next examined the localization of the wild-type and mutant Sli15 proteins in metaphase-arrested cells (Figure 6). As discussed above, both the mutant Sli15 proteins were present at similar levels to the wild-type protein. In the majority of metaphase-arrested cells, Sli15-EGFP was largely evident as a cloud of fluorescence surrounding the area of the metaphase spindle as previously reported [18], but with some Sli15-EGFP coincident with the spindle (Figure 6A). This was in contrast to the pattern shown by Sli15-20A-EGFP, which was tightly focused on the spindle and spindle poles in almost every metaphase arrested cell and which lacked the delocalized fluorescence surrounding the spindle seen in cells expressing wild-type Sli15-EGFP. This pattern of localization is essentially identical to that seen either when analog-sensitive Ipl1-as6 activity was inhibited or when an overlapping set of predicted Ipl1 phosphorylation sites in Sli15 were mutated to alanines (*sli15-17A*) [26]. In contrast, Sli15-20D-EGFP appeared to be completely delocalized in the metaphase-arrested cells, with greatly reduced levels associated with the spindle region in most cells. Quantitation of EGFP fluorescence coincident with spindle microtubules in all three strains confirmed these conclusions and demonstrated that the increased spindle association of Sli15 in the *sli15-20A* strain and the decreased levels in the *sli15-20D* strain were both highly statistically significant (Figure 6B). Thus non-phosphorylatable Sli15 showed increased metaphase spindle localization while the phosphomimic mutant showed reduced metaphase spindle localization, consistent with a role for Ipl1 phosphorylation of Sli15 in regulating its interaction with microtubules. Our novel finding that mimicking constitutive phosphorylation and blocking phosphorylation have opposite effects on spindle association of Sli15 *in vivo* strengthens the notion that its phosphorylation by Ipl1 regulates CPC localization and emphasizes that Sli15-20D has distinct properties in comparison with the non-phosphorylatable form.

**Figure 7:**
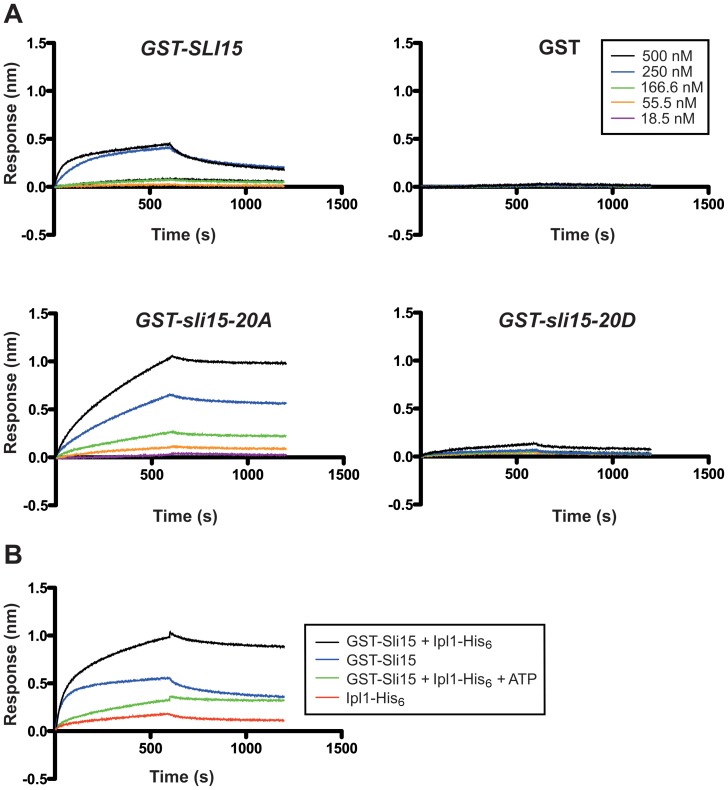
Binding kinetic analysis of wild type and mutant versions of Sli15 to immobilized microtubules by biolayer interferometry. A comparison of the binding abilities between recombinant GST-Sli15-His_6_ and its mutants to 5 µM taxol-stabilized, biotinylated polymerized tubulin, immobilized to streptavidin-coated biosensors is shown. (A) Binding results for five different concentrations of wild-type and mutant Sli15 (see inset) with equivalent concentrations of recombinant GST protein used as a control. Data shown are representative of three separate experiments. (B) Binding of wild type Sli15 and Ipl1 alone or in combination to taxol-stabilized, biotinylated polymerized tubulin with or without Sli15 phosphorylation by Ipl1 (+ATP); see inset for key. Data shown are representative of two separate experiments.

Since the behavior of the mutant Sli15 proteins *in vivo* strongly suggested that interaction of Sli15 with microtubules is affected by Ipl1 phosphorylation, we next investigated the direct binding of recombinant wild-type and mutant Sli15 proteins, prepared using GST/His_6_ tandem affinity purification, to taxol-stabilized microtubules. Using a novel assay based on biolayer interferometry, both GST-Sli15 and GST-Sli15-20A bound to microtubules, with GST-Sli15-20A showing greater binding over a range of concentrations (Figure 7). Thus even in the absence of phosphorylation, the wild-type protein binds microtubules less well than the alanine substitution mutant, showing that removal of the 20 polar side chains in the Sli15-20A microtubule domain can enhance its affinity for microtubules in comparison with the wild-type, non-phosphorylated protein. Most notably, however, binding of Sli15-20D was virtually undetectable even at the highest protein concentration used (500 nM). Thus the *in vitro* microtubule binding properties of the recombinant Sli15 proteins mirrored their behavior *in vivo* in metaphase-arrested cells and imply that the addition of multiple, negatively charged groups to the Sli15 microtubule-binding domain disrupts its ability to bind microtubules.

To confirm that the multiple amino acid substitutions in Sli15-20D were mimicking phosphorylation of Sli15 by Ipl1, we next compared the microtubule binding properties of GST-Sli15 in the presence of Ipl1, after pre-incubation either alone or with addition of ATP to allow Sli15 phosphorylation. Figure 7C shows that Ipl1 alone bound weakly to microtubules as expected from earlier studies [23], [45]. In the absence of ATP, GST-Sli15 and Ipl1 showed synergistic binding to microtubules. Strikingly, however, incubation with ATP caused a strong reduction in binding, indicating that phosphorylation of Sli15 by Ipl1 suppresses its affinity for microtubules. However, the phosphorylated complex still showed significant binding in comparison to Sli15-20D, possibly because the stoichiometry of phosphorylation was not maximal or because of additional effects of the Sli15-20D mutations.

### Sli15-20A and Sli15-20D show opposite patterns of localization along the anaphase spindle

**Figure 8:**
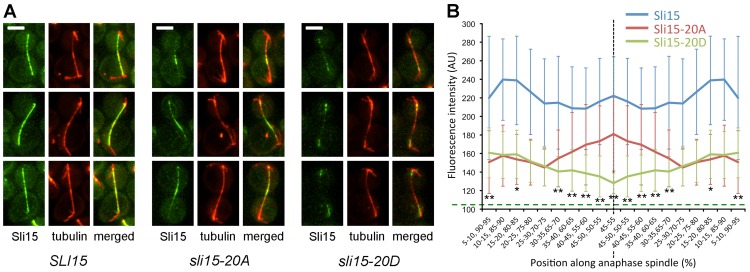
Localization of Sli15, Sli15-20A and Sli15-20D in anaphase cells. Metaphase-arrested cells of strains VMY357 (*SLI15*-EGFP), SJC591 (*sli15-20A*) and VMY375 (*sli15-20D*) prepared as in Figure 6 and allowed to progress into anaphase. (**A**) Representative images of anaphase cells from each strain (Sli15, Sli15-EGFP; tubulin, mCherry-Tub1). Bar, 3 µm. (**B**) Distribution of Sli15-EGFP along anaphase spindles in each strain. Sli15-EGFP fluorescence intensity along 12-16 anaphase spindles (6.0–12.6 µm) in each strain was measured on a normalized length scale of 0-100% and intensities sorted into 20 bins of increasing distance from the center of the spindle as shown, treating spindles as symmetrical. The mean fluorescence intensities in each region are plotted (error bars indicate standard deviation of the sample). Background fluorescence (∼100 arbitrary units; AU) was not subtracted but is indicated by the horizontal dashed line. The statistical significance of differences in the distribution of the Sli15-20A and Sli15-20D values in each bin was evaluated using the Mann-Whitney test (* = p<0.01; ** = p<0.001). The Sli15 wild-type profile was significantly different from the other two profiles at all points.

We next examined the localization of the three Sli15 proteins in cells allowed to progress from metaphase to anaphase (Figure 8). In anaphase cells expressing wild-type Sli15-EGFP the protein decorated the full length of the extended anaphase spindle in a punctate manner. However, in the equivalent *sli15-20A* strain, although Sli15 was clearly localized along the spindle, it frequently appeared more abundant in the central region. In contrast, Sli15-20D-EGFP appeared to be present at lower levels and was frequently absent from the central spindle. We quantitated this apparent difference by plotting the mean distribution of Sli15 fluorescence along multiple anaphase spindles from each of the three strains, finding a significant focusing of Sli15 in the central zone of the spindle in the *sli15-20A* spindles, compared with depletion of Sli15 in the corresponding region of the *sli15-20D* spindles (Figure 8). Thus in anaphase cells, Sli15-20A and Sli15-20D showed opposite patterns of distribution along the elongated spindle. Sli15-20D mimics constitutive phosphorylation of its Ipl1 sites, but is presumably dephosphorylated on its Cdk sites by Cdc14 phosphatase during anaphase. The bias in localization of Sli15-20D towards the poles of the anaphase spindle is reminiscent of the pattern seen when Sli15 remains phosphorylated on its Cdk sites (due to lack of Cdc14 phosphatase activity) but cannot be phosphorylated on its Ipl1 sites [26]. Thus phosphorylation of Sli15 may both reduce overall microtubule association and bias Sli15 localization towards the poles and away from the mid-zone of the anaphase spindle, with intermediate levels of phosphorylation on either the Ipl1 or Cdk sites being insufficient to prevent association.

### Strains lacking three distinct mechanisms for regulating the interaction of the CPC with spindle microtubules fail to show an additive growth defect

**Figure 9:**
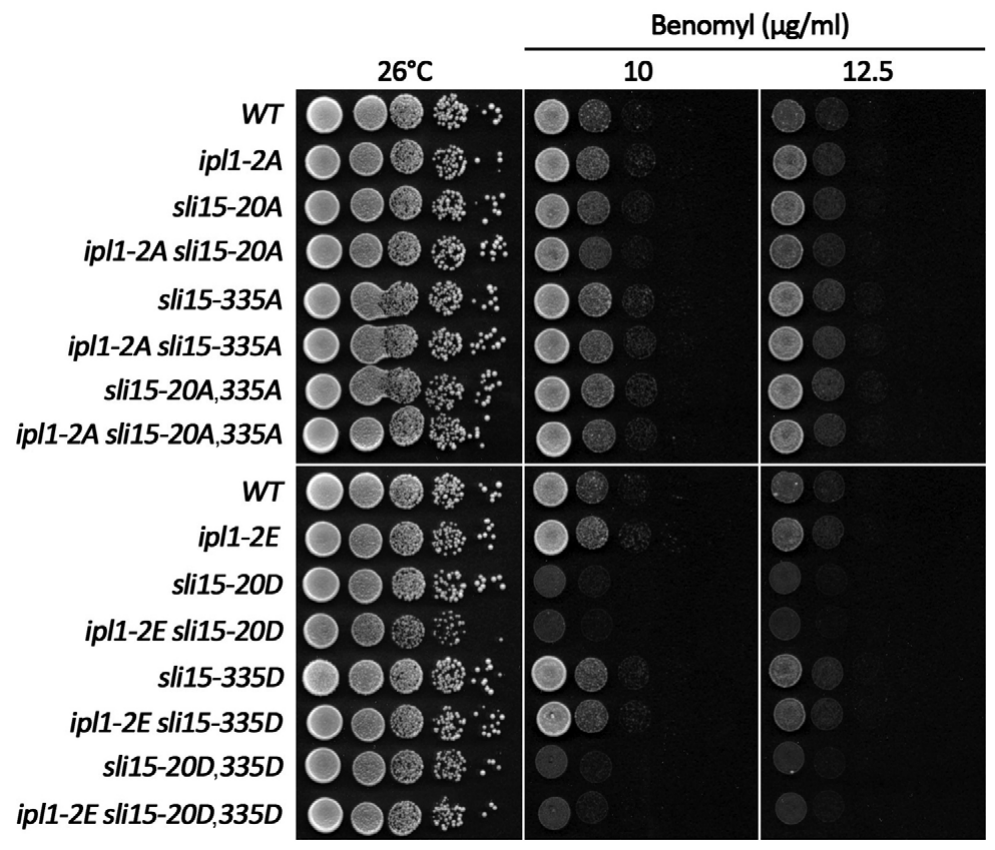
Strains lacking three distinct mechanisms for controlling the interaction of the CPC with spindle microtubules fail to show an additive growth defect. Equivalent 10-fold serial dilutions of the indicated strains were spotted onto YPAD medium and YPAD medium containing the indicated concentrations of benomyl and then incubated for 2 days at 26°C. *ipl1-2A* and *ipl1-2E* are alleles in which Cdc28 phosphorylation sites Ser-50 and Ser-76 in Ipl1 have been substituted by either alanine or glutamate residues respectively, while *sli15-335A* and *sli15-335D* have either alanine or aspartate substituted for the key serine residue in Sli15 that is phosphorylated by Cdc28 [18].

Recent work has indicated that several mechanisms influence the interaction of yeast CPC with microtubules including a weak, direct interaction between microtubules and Ipl1 itself [23], [45] that we have confirmed in our work, binding via Bim1 that is antagonized by Cdc28 phosphorylation on Ser-50 and Ser-76 in Ipl1 [45], Cdc28 phosphorylation of Sli15 (principally on Ser-335) that is antagonized by Cdc14 phosphatase as cells enter anaphase [18] and phosphorylation of Sli15 by Ipl1 [26] as shown here. Given the highly conserved nature of CPC relocalization to the spindle in anaphase, interfering with each of these pathways individually has surprisingly little impact on cell viability or proliferation, indicating that these mechanisms might function in a redundant manner to regulate CPC localization. We therefore attempted to generate strains containing different combinations of either alanine or phosphomimic substitution mutations affecting the three phosphorylation-dependent mechanisms. However, all combinations of mutations could be generated without any signs of synthetic negative genetic interactions, both when the mutations would be expected to drive premature microtubule binding or to interfere with it (Figure 9). Thus while each of these mechanisms leads to detectable phenotypes when perturbed, even in combination such perturbations still have limited consequences for proliferation.

## Discussion

### Identification of Sli15 phosphorylation sites

It has been known for some time that INCENP/Sli15 shows Aurora B/Ipl1-dependent phosphorylation [24], but apart from the implication of IN-box phosphorylation in full Aurora B kinase activation [23], the role of Aurora B/Ipl1-dependent phosphorylation elsewhere in INCENP/Sli15 has been unclear. Here we have identified fourteen specific residues in Sli15 that are phosphorylated *in vitro* by Ipl1, eleven of which fall within the central domain of the protein that has been implicated in microtubule binding, and all of which are upstream of the conserved IN-box required for Ipl1 kinase activation. The majority of the sites we have mapped fall within the accepted consensus for Aurora B/Ipl1 phosphorylation [13], but two of the sites we identified support the notion that asparagine is also a preferred residue at +1, in keeping with a recent survey of Aurora B phosphorylation sites [37], and the consensus for Aurora B/Ipl1 phosphorylation should therefore be adapted to include asparagine at the +1 position i.e. [KR]-X-[ST]-[ILVSTN]. Six of the sites we identified *in vitro* have been found to be phosphorylated *in vivo*, and only two sites conforming to the accepted consensus that are now known to be phosphorylated *in vivo* were not identified in our study. Our work therefore strengthens the link between these six *in vivo* phosphorylation sites and Ipl1 by demonstrating that the kinase can phosphorylate them directly, and that it is therefore likely to be the *in vivo* kinase. None of our phosphorylation sites are located within the IN-box, consistent with our finding that non-phosphorylatable Sli15 (Sli15-20A) can promote full activation of Ipl1 in an *in vitro* protein kinase assay using Dam1 as a substrate. Thus direct activation of Aurora B/Ipl1 via IN-box phosphorylation may not be a conserved feature of the CPC, consistent with the apparent lack of conservation of the activatory phosphorylation sites in the IN-box region [23] in many lower eukaryotes. Conversely, multiple consensus sites for Aurora B/Ipl1 are found in the central domain of INCENP/Sli15 from a wide variety of organisms including mouse, chicken, slime moulds and yeasts (not shown), supporting the notion that phosphorylation of this region by Aurora B/Ipl1 may represent a conserved feature of the CPC.

### Sli15 phosphorylation by Ipl1 is not required for chromosome biorientation

Yeast cells relying on either non-phosphorylatable or phosphomimic alleles of *SLI15* were fully viable, in contrast to cells in which conditional mutations in either the *SLI15* IN-box region [36] or *IPL1* causes lethality due to failed chromosome biorientation under restrictive conditions [2]. This implies that both *sli15-20A* and *sli15-20D* strains should be capable of promoting efficient chromosome biorientation. Consistently, we could detect no significant change in the efficiency of chromosome biorientation in either strain, in agreement with the properties of a similar non-phosphorylatable *sli15* mutant [26]. Our data therefore indicate that constitutive phosphorylation of Sli15 on the Ipl1 sites is unlikely to interfere with chromosome biorientation despite the benomyl hypersensitivity of the *sli15-20D* strain. The robust spindle association of Sli15-20A in pre-anaphase cells could potentially reduce kinetochore-localized CPC, but efficient chromosome biorientation in the *sli15-20A* strain indicates that Ipl1 can still gain proper access to its substrates on incorrectly attached kinetochores. Deletion of the entire N-terminal domain of Sli15, which mediates its association with the other CPC components (Bir1 and Nbl1) involved in centromere targeting, also drives Sli15 onto the pre-anaphase spindle but has little or no effect on the efficiency of chromosome biorientation or chromosome segregation [8]. While this questions the importance of centromere targeting of the CPC at least in yeast, it underlines the view that an abnormal CPC association with the pre-anaphase spindle is not an obstacle to achieving efficient chromosome biorientation.

### Sli15 phosphorylation by Ipl1 affects its interaction with spindle microtubules

Phosphorylation of the central domain of Sli15 on its cyclin-dependent kinase (Cdk) sites is known to regulate its interaction with spindle microtubules, and alanine substitution of either just ser-335 or all six Cdk phosphorylation sites drives Sli15 onto the spindle prematurely in metaphase-arrested cells [18]. Both our non-phosphorylatable Sli15-20A protein and another similar Sli15 mutant [26] show strong localization to the metaphase spindle that mimics the effect of inhibiting Ipl1 [26], indicating that phosphorylation by Ipl1 is involved in restricting interaction of the CPC with the spindle in pre-anaphase cells. Consistent with its reduced spindle localization *in vivo*, Sli15-20D was completely defective in binding microtubules *in vitro* whereas Sli15-20A and wild-type Sli15 bound microtubules well. Binding of the wild-type protein was strongly reduced following phosphorylation by Ipl1, indicating that phosphorylation of Sli15 by Ipl1 directly affects its affinity for microtubules and confirming that addition of multiple negative charges to the microtubule domain is inhibitory to binding. The opposite behavior of Sli15-20A and Sli15-20D in relation to microtubule association is therefore in contrast to alanine or aspartate substitutions at the six Cdk phosphorylation sites in Sli15, both of which conferred similar behavior [18]. This indicates that Sli15-20D is not just behaving as non-phosphorylatable form of Sli15 but that it has distinct properties conferred by the negatively-charged side chains of the glutamate and aspartate residues. Thus the poor *in vitro* microtubule binding properties of both Sli15-20D and of phosphorylated wild-type Sli15 in comparison with the non-phosphorylated protein or Sli15-20A support the contention that Sli15-20D is mimicking the constitutively phosphorylated form of the protein. Given that neither *sli15-20A* nor *sli15-20D* confer a significant chromosome biorientation defect, it is likely that the elevated chromosome loss rates observed in each mutant result from altered behavior of the spindle microtubules resulting from inappropriate levels of CPC association, although we cannot exclude the possibility of a small defect in bi-orientation that was below the limit of detection in our biorientation assay.

Since it is likely that Sli15-20A remains phosphorylated on its Cdk sites, Cdk phosphorylation on its own may be insufficient to block spindle association of the CPC. However, since Sli15-20D retains some ability to interact with the anaphase spindle, albeit at a reduced level, Ipl1 phosphorylation also appears insufficient to block spindle association of the CPC in anaphase when the Cdk sites have been dephosphorylated. Thus our data and those of Nakajima *et al.* [26] support the notion of combinatorial regulation of CPC localization by both Ipl1 and Cdk phosphorylation. While phosphorylation at the Cdk sites in Sli15 is clearly regulated, it is not clear to what extent phosphorylation of the Ipl1-dependent sites may also be controlled. Sli15 bound to Ipl1 within the CPC might be constitutively phosphorylated on its Ipl1 sites, providing a constant level of phosphorylation to tune the basal microtubule binding affinity of the complex and against which changes in phosphorylation of the Cdk sites can push the affinity for microtubules in one direction or the other. Ipl1 is clearly active throughout anaphase [46], supporting the idea that Sli15 may remain phosphorylated on its Ipl1 sites at this stage, but protein phosphatases such as PP1, which is known to counteract Ipl1 function [41], [47], may provide a means to antagonize CPC autophosphorylation.

Association of the CPC with spindle microtubules is regulated in multiple ways: binding via the interaction between Ipl1 and Bim1 is inhibited by Cdk phosphorylation on Ser-50 and Ser-76 [45], while both Cdk and Ipl1-dependent phosphorylation of Sli15 antagonize its interaction with microtubules [18], [26] as shown in our study. It therefore seemed likely that these different mechanisms would cooperate to regulate spindle association of the CPC, since blocking any of these sets of regulatory phosphorylations can drive premature spindle association of the CPC. It is therefore remarkable that interfering with all three pathways concurrently failed to confer any obvious additive defect. Perhaps additional redundant pathways await discovery or else the functions conferred by modulating spindle localization represent fine tuning mechanisms, improving the efficiency of chromosome segregation but not an essential component of it. Given the conservation of CPC relocalization at the metaphase to anaphase transition it is surprising that neither the conserved mechanisms governing its interaction with centromeres [8] nor the multiple regulatory pathways that control its spindle localization [18], [26], [45] appear to be essential.

### Sli15 phosphorylation by Ipl1 and the tension checkpoint

Our work has shown that in contrast to the *sli15-20A* mutant, strains dependent on Sli15-20D fail to delay entry into anaphase in response to reduced tension on kinetochore-microtubule attachments – the ‘tension checkpoint’. However, both *sli15* mutants showed a normal metaphase arrest in response to microtubule depolymerisation by nocodazole, indicating that the core checkpoint machinery is functional. Ipl1 [39] and other components of the CPC [31], [48] are required specifically for the tension checkpoint response and our data suggest that phosphorylation of Sli15 by Ipl1 may negatively regulate the yeast cell's ability to sense tension on microtubule-kinetochore connections. Previous work demonstrated that the CPC components Bir1 and Sli15 can form a physical link *in vitro* between centromeric DNA and microtubules that requires the central microtubule-binding domain of Sli15 and that is inhibited by Ipl1 phosphorylation [36]. This linkage was proposed to be a tension sensor for activation of Ipl1 leading detachment of incorrect microtubule attachments so as to promote chromosome biorientation, although more recent work has favored models whereby tension regulates biorientation via spatial separation of the CPC from its kinetochore substrates [49]. Our finding that Sli15-20D confers a profound defect in the tension checkpoint without preventing efficient chromosome segregation is therefore consistent with a role for Sli15 and its interaction with microtubules in tension sensing, but in the context of spindle assembly checkpoint signaling rather than the promotion of chromosome biorientation; phosphorylation of Sli15 by Ipl1 may therefore act as a negative regulator of the tension checkpoint.
